# Antibiotic resistance increases evolvability and maximizes opportunities across fitness landscapes

**DOI:** 10.3389/fmicb.2026.1905746

**Published:** 2026-09-18

**Authors:** Fabrizio Spagnolo, Daniel E. Dykhuizen

**Affiliations:** 1 Department of Natural and Life Sciences, Long Island University-Post, Brookville, NY, United States; 2 Department of Ecology and Evolution, Stony Brook University, Stony Brook, NY, United States

**Keywords:** antibiotic resistance, compensatory mutation, evolvability, experimental evolution, fitness landscape

## Abstract

Antibiotic resistance continues to grow as a public health problem. One of the reasons for this continued growth is that resistance to antibiotics is strongly selected for in the presence of antibiotics and weakly selected against after their removal. This is frequently thought to be due to the effects of compensatory mutations. However, compensatory mutations are often not found in clinically relevant strains of antibiotic resistant pathogens. Here, we conduct experiments *in vitro* using chemostats and serial transfer flasks that highlight the role that fine scale differences in environment play in the maintenance of populations after selection for resistance. Chemostat conditions resulted in a different set of mutations than in flasks, showing that the differences in the mode of growth are capable of reliably changing the force and direction of selection. Our results suggest that antibiotic resistance can make novel adaptive paths available to populations through newly arising variation, allowing populations to climb previously unavailable adaptive peaks in a manner similar to Wright’s shifting balance, but driven only by selection.

## Introduction

One of the most difficult aspects of the antibiotic resistance problem is the contrast between the speed and strength of selection *for* antibiotic resistant strains and the slower selection *against* resistant strains of bacteria (and for reversion to antibiotic sensitivity). The evolution for antibiotic resistance occurs either due to *de novo* mutations or through horizontal gene transfer events. The concentration of antibiotics in the environment can either be low enough to allow the sensitive strains to survive but high enough to select for resistant strains or the concentration of antibiotics in the environment can be high enough that the drug susceptible phenotypes die off/stop growing, leaving only resistant phenotypes to survive, grow, and reproduce. Different mutations are selected in these two cases (Wistrand-Yuen et al., 2018). In this paper we are studying the latter case, where only the resistant strains survive because of high levels of antibiotics. Essentially, this is the strongest way that selection can possibly act: either you are susceptible and quickly die, or you are resistant and survive [neglecting persisters (Balaban et al., 2019) for the time being]. In laboratory experiments, the evolution of a population from susceptible to resistant has been shown to occur on the scale of hours to days (Toprak et al., 2011; Miller et al., 2013; Spagnolo et al., 2016).

As frequencies of antibiotic resistance rose worldwide, coordinated attempts were undertaken to reverse resistance by removing the environmental conditions that promoted it in the first place (Ridley et al., 1970). In essence, the strategy was to select against resistance as quickly and consistently as possible. This was done primarily by lowering rates of antibiotic usage (Luyt et al., 2014) and relying on the phenomenon known as the “cost of resistance” (Levin et al., 1997; Andersson and Levin, 1999; Austin et al., 1999), whereby many resistant strains have lower fitness than the sensitive strains in the absence of antibiotics. Typically, this cost occurs because a vital phenotype, such as protein or membrane synthesis, is the target of the antibiotic, and therefore of the resistance mechanism as well. As such, the phenotype of resistant mutants is not like wild type, making them less fit in direct competition. Results using this ecological approach have been mixed (Andersson and Hughes, 2010), with reversion away from resistant phenotypes and back to antibiotic sensitivity shown to occur slowly (Seppala et al., 1997), quickly (van den Bogaard et al., 2000; Agersø and Aarestrup, 2013), or not at all (Smith, 1975; Mölstad et al., 2008).

Resistant mutations are reliably selected for when antibiotics are used for medical or agricultural purposes (Bush et al., 2011; The Review on Antimicrobial Resistance, 2016), but do not always decline in frequency when the antibiotics are removed from the environment. It has been proposed that this is due to compensatory mutations (Maisnier-Patin and Andersson, 2004). Compensatory mutations relieve the cost of resistance through partial restoration of the impacted phenotype (Reynolds, 2000; Szamecz et al., 2014; Hughes and Andersson, 2017) and have been consistently found in laboratory experiments (Schrag et al., 1997; Reynolds, 2000; Maisnier-Patin and Andersson, 2004). But, compensatory mutations are often absent from clinical strains of antibiotic resistant bacteria (Andersson and Levin, 1999; MacLean and Vogwill, 2015), suggesting that something more is at play.

In order better understand post-resistance adaptation in bacterial populations, we conducted several investigations using streptomycin resistant strains of *Escherichia coli* with a K42N mutation in ribosomal protein S12 under *in vitro* conditions with controlled differences between them. The results indicate that the adaptive landscape available to a population is of vital importance to how the population evolves, even when environmental differences seem to be of extremely fine scale, suggesting that how populations adapt in antibiotic resistance is even more intricate than previously feared.

## Methods

### Strains

All bacterial strains used are *E. coli* K-12 MG1655 strains. The ancestral strain for all subsequent experiments is labeled as DD1953 and is known to be free of any plasmids, to be antibiotic sensitive, and to be *rpoS-* due to a premature stop codon in the coding sequence of the *rpoS* gene. A *rpoS-* strain was chosen so as to not complicate our results with the possibility of stress responses resulting from near-starvation of the cells in the chemostat as opposed to the temporal variability of the limiting nutrient in serial dilution trials. The DD1953 strain served as the streptomycin sensitive ancestral strain for all experiments. In order to assure genetic homogeneity at the start, DD1953 was grown from a single *E. coli* colony from an agar plate, grown in liquid media and then frozen for long term use across all experiments described here.

To generate streptomycin resistant mutants of DD1953, we plated 20 mL of overnight DD1953 into 200 mL of fresh LB agar (at 42 °C) containing 100 μg/mL of streptomycin. A streptomycin resistant mutant that grew in the agar was then collected. The single nucleotide polymorphism (SNP) granting streptomycin resistance was identified by MiSeq and confirmed by Sanger DNA sequencing to be a nonsynonymous change in the *rpsL* gene at codon 42, changing the wild type lysine to asparagine (K42N). A pure freezer stock was prepared and stored at −80 °C. This K42N *rpsL* mutant is the direct ancestor of all subsequent experimentally evolved populations. This resistant ancestor is labeled as FS1.

A mutant of FS1 that was also resistant to bacteriophage T5 was then generated via another mutant screen and labeled as FS5. Resistance to the bacteriophage T5 was used as a molecular marker in experiments where one was needed, such as in direct competition experiments. Resistance to T5 is due to a SNP in the *fhu* gene and has long been known to be selectively neutral in chemostat experiments (Moser, 1958). The minimum inhibitory concentrations (MIC) for all strains were determined experimentally using methods identical to those described earlier (Spagnolo et al., 2016). We found that DD1953 had a MIC for streptomycin of 2–4 μg/mL, with all resistant strains having MICs over 1,000 μg/mL.

### Chemostats and serial transfer flasks

To inoculate experimental chemostats and flasks, we grew strains FS1 and FS5 inoculated from frozen cultures overnight in flasks containing Davis salts and 0.1% glucose at 37 °C shaken at 200 rpm. These cultures were diluted the next morning into fresh media and the growth followed by measuring optical density on a Biowave CO8000 cell density meter (Harvard Bioscience, Holliston, MA). When the OD_600_ reached 0.7, we added 1 mL of each inoculating strain to chemostats and/or flasks containing 30 mL of Davis salts supplemented with 0.01% glucose (w/v) as the only available carbon source. Streptomycin concentrations for all experimental evolution vessels were as indicated.

All continuous culture experiments were conducted in Kubitschek chemostats following procedures previously described (Dykhuizen, 1993). The generation time was set to 3.69 h per generation (6.5 gen/day, D = 0.1875), matching the mean number of generations per day in the 1:100 dilution of flask cultures (Lenski et al., 1991). Additional chemostat related methods are in the Supplementary material. A total of 4 chemostats were run simultaneously. Chemostats 1 & 2 utilized the Davis minimal media with streptomycin at a concentration of 100 μg/mL (high streptomycin condition). Chemostat 3 had the Davis minimal media and 16 μg/mL of streptomycin (low streptomycin condition) as per Miller (1992). Chemostat 4 had Davis minimal media but contained no streptomycin (no streptomycin condition). All streptomycin (Sigma-Aldrich, St. Louis, MO) came from a single prepared stock (10 mg/mL).

Two serial transfer flask experiments were run concurrently with the chemostat experiments. Whereas the chemostats maintained the experimental populations in a constant state of sub-maximal exponential growth, the serial transfer flask populations underwent full cycles of lag phase, maximal exponential growth, and stationary phase each day. Two sterile 250 mL flasks were prepared with 30 mLs of fresh Davis minimal media, 0.01% glucose, and 100 μg/mL of streptomycin (high streptomycin condition). The flasks were placed in an incubator, maintained at 37 °C and constantly shaken at 200 rpm for 24 h. After a 24-h period, 300 μL of the experimental sample was transferred into a fresh 250 mL flask with 30 mL of fresh media. The 100-fold dilution regime and 24 h time frame allows for the calculated number of generations to be 6.5 generations, as per Lenski et al. (1991). This experimental design allows the *average* generation time of the *E. coli* in the flasks to match the *maintained* generation time in the chemostats.

### Whole-genome sequencing

Miseq next-generation sequencing (Illumina, San Diego, CA) was performed on population samples. Libraries were prepared using the Illumina TruSeq genomic DNA-nano kit and sequenced using Illumina MiSeq 2×300 protocols at the University of Wisconsin Biotechnology Center (Madison, WI). Overall mean coverage of the genomic sequencing was 162X (SD: 28). Fastq files containing all reads are archived in SRA at NCBI (BioProject PRJNA1499252).

### Bioinformatics

All bioinformatics was performed in the Galaxy environment (The Galaxy Community, 2022). Raw sequence reads were vetted for quality using FastQC and aligned to the ancestral genome (DD1953) using the reference K-12 MG1655 genome as a scaffold (Genbank accession NC_000913.3). SNP calling and indel identification were conducted via the VarCap workflow (Zojer et al., 2017), which also provides SNP frequency data.

### Protein synthesis assay

Streptomycin acts by binding near the A-site of the ribosome, interfering with the function of protein S12, the product of the *rpsL* gene. The mechanism of action for streptomycin is interfering with protein synthesis efficiency, both in rate of protein synthesis and fidelity (Kurland, 1992). Structural changes to the S12 protein, particularly at amino acid position 42, are known to confer high level resistance to streptomycin, but result in reduced protein synthesis efficacy (Bohman et al., 1984; Allen and Noller, 1989; Agarwal et al., 2011).

In order to compare protein synthesis rates for streptomycin sensitive, resistant, and evolved strains, we performed a protein synthesis assay. S30 cell lysate was extracted from an exponentially growing population of *E. coli* that was to be assayed. The S30 lysate extraction protocol was based upon that of Shrestha (Shrestha et al., 2012) with some modification (See SI). Following S30 lysate extraction, a firefly luciferase assay was performed as per the *E. coli* S30 Extract System for Circular DNA by Promega guidelines for control experiments using the Promega pBEST*luc* plasmid for *E. coli* (Promega, Madison, WI). Following 60 min of incubation at 37 °C, relative light units (RLU) were measured in triplicate using a Turner Biosystems (Sunnyvale, CA) Model 20/20n luminometer (See SI).

### Fitness assay

In order to compare relative fitness of resistant and evolved strains, direct competitions between strains were conducted in chemostats in the manner of Dykhuizen (Dykhuizen and Hartl, 1980; Dykhuizen, 1993). Using T5 bacteriophage resistance as a molecular marker, two strains were inoculated into a chemostat in equal numbers. Changes in the frequencies of these strains were tracked over 72–96 h via plating as described (SI).

By plotting the natural log of the ratio of mean CFUs per unit time, the coefficient of selection was obtained from the slope of the plotted trendline (Dykhuizen, 1993). From these measures, the relative fitness measure, w_R_, was calculated by use of the equation:


$$w_{R}=e^{s}$$

All chemostat parameters were as described but the concentration of streptomycin was varied to coincide with the context of the competition. We note that competition was under chemostat conditions even for strains evolved in flasks.

### Growth curve assay

We obtained growth curve data for strains by monitoring optical density (*λ* = 600 nm) in 96-well plates in a SpectraMax Model 384 Plus microplate reader using SoftMaxPro software (Molecular Devices, Sunnyvale, CA) over a ten-hour period. Briefly, we grew strains overnight in 3 mL of LB (rich media). The next day, 300 μL of the overnight growth was added to 9 mL of Davis minimal media (1:30 dilution). The media-strain mixture was vortexed to insure even distribution. The inoculated media was then put into replicate wells on the 96 well plate, with 180 μLs in each well and at least 1 negative control well per set of technical replicates. A minimum of 16 replicates were run for each sample.

Following inoculation, the 96 well plate was placed into the plate reader and the populations were allowed to grow at 37 °C. The OD was measured every 60 s over a period of 10 h, with mixing of the plate between reads. Typically, the populations reached stationary phase well before the 10-h mark. Data was analyzed using the grofit package (Kahm et al., 2010) and R statistical software. The grofit package determines the time point for transition into exponential growth as well as into stationary phase, thereby allowing for analysis of length of time spent in lag and exponential growth phases, as well as the maximum growth rate (μ_max_), and the density at the transition into stationary phase. The amount of time spent in exponential growth was calculated by finding the difference between the time point where a well population entered stationary phase and the time point where exponential phase was entered.

## Results

Based upon the results of our fitness assays as well as the substantial body of previous work regarding mechanisms of fitness increases (Schrag and Perrot, 1996; Schrag et al., 1997; Hughes and Andersson, 2017; Balaban et al., 2019) showing fitness increasing mechanisms related to target modification, antibiotic inactivation, and efflux, particularly with regard to streptomycin resistance, we expected our experimental results would confirm previous work. Our presumption was only partially correct.

### Loss of fitness in streptomycin resistant mutants

Amino acid changes at position 42 of the ribosomal protein S12 confer resistance to high doses of streptomycin. This mutation also confers substantial fitness costs in *Escherichia coli* (Kurland, 1992), *Mycobacterium tuberculosis* (Sander et al., 2002) and *Salmonella enterica* (Bjorkman et al., 1998). When two *E. coli* strains with spontaneous streptomycin resistant mutations (both mutations were in codon 42 in the *rpsL* gene) were individually competed against an isogenic susceptible strain in flask cultures of a modified Davis salts medium with limited glucose (Schrag and Perrot, 1996; Schrag et al., 1997), the coefficients of selection were 0.11–0.19 against the resistant strain.

Using a streptomycin resistant mutation in *Salmonella typhimurium* LT2 [K42N in the S12], Paulander, *et al.* measured maximal growth rate in pure culture and then compared growth rates of the strep-resistant strain to the sensitive strain to determine relative fitness (Paulander et al., 2009). The sensitive strain grew faster on a rich media (LB) and on M9 minimal media (MM) supplemented with glucose or with glycerol, but grew more slowly on poor media—M9 MM supplemented with pyruvate or with succinate. They showed this was due to the differential induction of the stress-inducible sigma factor, RpoS. On the poor media the strep-sensitive cells induce *rpoS*, which retarded growth. When both cells lack *rpoS*, the effect disappears. This example shows how the effects of the environment and other physiological systems in the cell can reverse expectations.

We competed streptomycin resistant mutants, FS1 and FS5 (Table 1) in glucose limited chemostats. These mutations were in codon 42 of the S12 ribosomal protein, just as in the above example (Paulander et al., 2009). Strains FS1 and FS5 had lower relative fitness when compared to the streptomycin sensitive ancestor, DD1953. The fitness of FS1 (T5 bacteriophage susceptible) and FS5 (T5 bacteriophage resistant) was measured to be 0.91 ± SD 0.02 relative to DD1953 (mean of three samples) in our fitness assay (Table 2). This level of fitness loss is in line with that found in previous experiments (Schrag and Perrot, 1996; Björkman et al., 2000).

**Table 1:** Strain names and experimental conditions.

	Strain name	Description	Growth condition	Streptomycin concentration
Antibiotic sensitive ancestor	DD1953	wild type ancestor: 2 ≥ MIC ≤ 4		
Antibiotic resistant ancestors	FS1	resistant ancestor strain (*rpsL*: K42N)		
	FS5	resistant ancestor strain (*rpsL*: K42N) T5 bacteriophage resistant		
Evolved antibiotic resistant strains	4C2-12R	Experimentally evolved	Chemostat	High
	4C3-10S	Experimentally evolved	Chemostat	Low
	4C4-10S	Experimentally evolved	Chemostat	No Streptomycin
	4S1-44R	Experimentally evolved	Serial Transfer	High
	4S1-44S	Experimentally evolved	Serial Transfer	High
	4S2-44S	Experimentally evolved	Serial Transfer	High

All strains were derived from a single antibiotic sensitive ancestor, DD1953. Two streptomycin resistant mutants of DD1953 were generated, FS1 and FS5, differing only in resistance to T5 bacteriophage, which was used as a neutral molecular marker. Evolved strains experienced different in vitro environments (4C designates chemostat evolved and 4S means evolved in serial batch culture). Chemostat evolved strains also had a range of set streptomycin concentrations for their time under experimental conditions (last column).

**Table 2:** Strain relative fitnesses before and after evolution experiment.

Strain	Fitness
Pre-experiment	
DD1953	1
FS1	0.919
FS5	0.917
Post-experiment	
FS1	1
FS5	0.998
4C2-12R	1.04
4C3-10S	1.03
4C4-10S	1.03
4S1-44S	1.02
4S1-44R	1.02
4S2-44S	ND

All relative fitnesses were quantified from direct competition in chemostats. Fitness was measured as the slope of the best fit line for a time series of ratios of T5 sensitive to T5 resistant colonies (Dykhuizen and Hartl, 1980). A. Fitnesses of streptomycin resistant ancestors (FS1, FS5) relative to their streptomycin sensitive ancestor, DD1953. B. Relative fitnesses of all evolved strains relative to their pre-experiment ancestor, as obtained by direct competition. ND indicates not determined.

### Fitness increases via compensatory mutations in batch culture

As in previous experiments with streptomycin resistant bacteria in batch culture, the streptomycin resistant mutants rapidly selected for compensatory mutations in the *rpsD* and *rpsE* genes (S4 and S5 small ribosomal proteins, respectively) (Table 3) and increased in fitness relative to the ancestor (2–4%). The compensatory mutations did not affect MICs of our strep-resistant strains.

**Table 3:** Genetic changes in evolved strains relative to antibiotic resistant ancestor.

Evolved strain	Gene and change	Frequency	Description
4C2-12R	*galS* Deletion	0.14	1,022 bp deletion
	*galS* Deletion	0.26	1 bp deletion
	*malT*	0.41	Nonsynonymous change
	*malK*	0.43	Nonsynonymous change, ATP-binding subunit
4C3-10S	*galS*	1	Nonsynonymous change
4C4-10S	*galS*	1	Synonymous change
4S1-44R	*rpsD*	0.27	Structural constituent of ribosome; nonsynonymous change, L198P. Previously unreported compensatory mutation.
	*rpsD*	0.2	Structural constituent of ribosome; nonsynonymous change, I199S. Previously unreported compensatory mutation.
	*rpsE*	0.5	Structural constituent of ribosome; nonsynonymous change, G108V. Known compensatory mutation site.
	*acs*	0.6	Acetyl CoA synthase, acetate-scavenging enzyme, improves carbon starvation survival; promoter region
4S1-44S	*rpsE*	1	Structural constituent of ribosome; nonsynonymous change, G108A. Known compensatory mutation site.
	*yjcO* intergenic region	0.27	Sel1 family TPR-like repeat protein; function unknown
4S2-44S	*gstB* promoter region	1	glutathione-dependent bromoacetate dehalogenase
	*rpsE*	1	Structural constituent of ribosome; nonsynonymous change, G86C. Known compensatory mutation.
	*yjcO* intergenic region	0.26	Sel1 family TPR-like repeat protein; function unknown
	*yjeJ* proximal promoter region	1	Uncharacterized protein

Genetic changes identified through MiSeq relative to the relevant antibiotic resistant ancestor (FS1 or FS5). SNP frequency, as quantified through the VarCap bioinformatics pipeline, as well as function data (as per the Ecogene database, http://ecogene.org) are provided for each mutated gene.

Ribosomal ambiguity mutations (ram) in *rpsD* and *rpsE* have been shown to be compensatory (Maisnier-Patin et al., 2002) for hyperaccurate *rpsL* mutants, such as ours. This is believed to be because of ram mutants’ pleiotropic effect of increasing polypeptide synthesis rates, thereby counterbalancing the increased synthesis time caused by slowed proofreading in hyperaccurate mutants of *rpsL* (Bohman et al., 1984; Poehlsgaard and Douthwaite, 2005; Holberger and Hayes, 2009). These ram mutations do not increase polypeptide synthesis when the genetic background includes a wild type *rpsL* gene, as measured *in vitro* using radioactive isotope tagged amino acids (Andersson et al., 1982).

There are two common approaches to testing the overall fitness effects of compensatory mutations such as these. While we utilize the second, the first taken by Schrag and Perrot, warrants mention (Schrag and Perrot, 1996). They used P1 to transduce both the streptomycin sensitive and the streptomycin resistant alleles into the evolved cultures carrying the streptomycin resistant mutation. Their results were quite variable but in all cases the strain carrying the resistant allele had an advantage over the strain carrying the sensitive allele in flask cultures. They found compensatory mutations in the *rpsD* and *rpsE* genes, which were selected for subsequent to changes at position 42 in the S12 ribosomal protein, but these *rpsD* and *rpsE* mutants were *detrimental when paired with the wild type streptomycin sensitive allele*. Put differently, these compensatory mutations led to a condition where reversion to streptomycin sensitivity would be *selected against*, highlighting that genetic background matters in determining the fitness of the strain carrying a mutation giving resistence to streptomycin.

To investigate compensatory mutation fitness effects in our experiments, the evolved strains carrying the mutation giving streptomycin resistance were competed against unevolved strains carrying the streptomycin resistance allele. The evolved strains had all been evolved either in flask culture or in chemostats (Table 1). Overall, evolved strains increased fitness relative to their streptomycin resistant ancestors 2–4% above where they started in the experiments (Table 2).

### No previously identified compensatory mutations in chemostats

In our chemostat experiments, the growth medium was identical to that of the flask experiments, although the growth conditions clearly were not. We also varied the concentration of streptomycin in different chemostats to test for any effect. The general results were that fitnesses increased in these evolved strains, but the genetic mechanism was different. In chemostat cultures, we found no compensatory mutations in *rpsL, rpsE,* or *rpsD* (Table 3). By performing whole-genome sequencing of evolved clones, we identified changes in the glucose uptake systems of the chemostat evolved strains, particularly in *galS*. Similar genetic changes were previously observed in chemostat evolved strains of strep-sensitive *E. coli* (Notley-McRobb and Ferenci, 1999), where changes to the Mgl/GalS system improved uptake of glucose across the inner membrane when the glucose was at low concentration. This previous result suggests that the changes are not unique to streptomycin resistant mutants (and are thus not compensatory), but more likely adaptations to growth under chemostat conditions.

### Unexpected phenotypic changes

We performed growth curve assays in order to better understand the nature of the observed fitness increases. The expectation was that we would observe increases in maximum growth rates in the flask-evolved strains as a result of the compensatory mutations increasing protein synthesis efficiency. However, even prior to the experimental evolution experiments, maximum growth rates did not differ between strep-sensitive DD1953 and the resistant clones FS1 and FS5 in minimal glucose media. In the evolved strains, we were surprised to find no discernible pattern of increase in maximal growth rates, regardless of whether strains evolved in flasks or chemostats (Figure 1B). We note that the low maximum growth rate of strain 4C2-12R, which could be because of some unknown mutational or regulatory change, seems to be reflected in longer time in exponential growth. This could be a unique phenomenon for which we have no genetic explanation. Additionally, there was no observable pattern to changes in time spent in lag phase or the population density at entry to stationary phase for evolved strains when compared to the strep resistant ancestors (Figures 1A,C).

**Figure 1:**
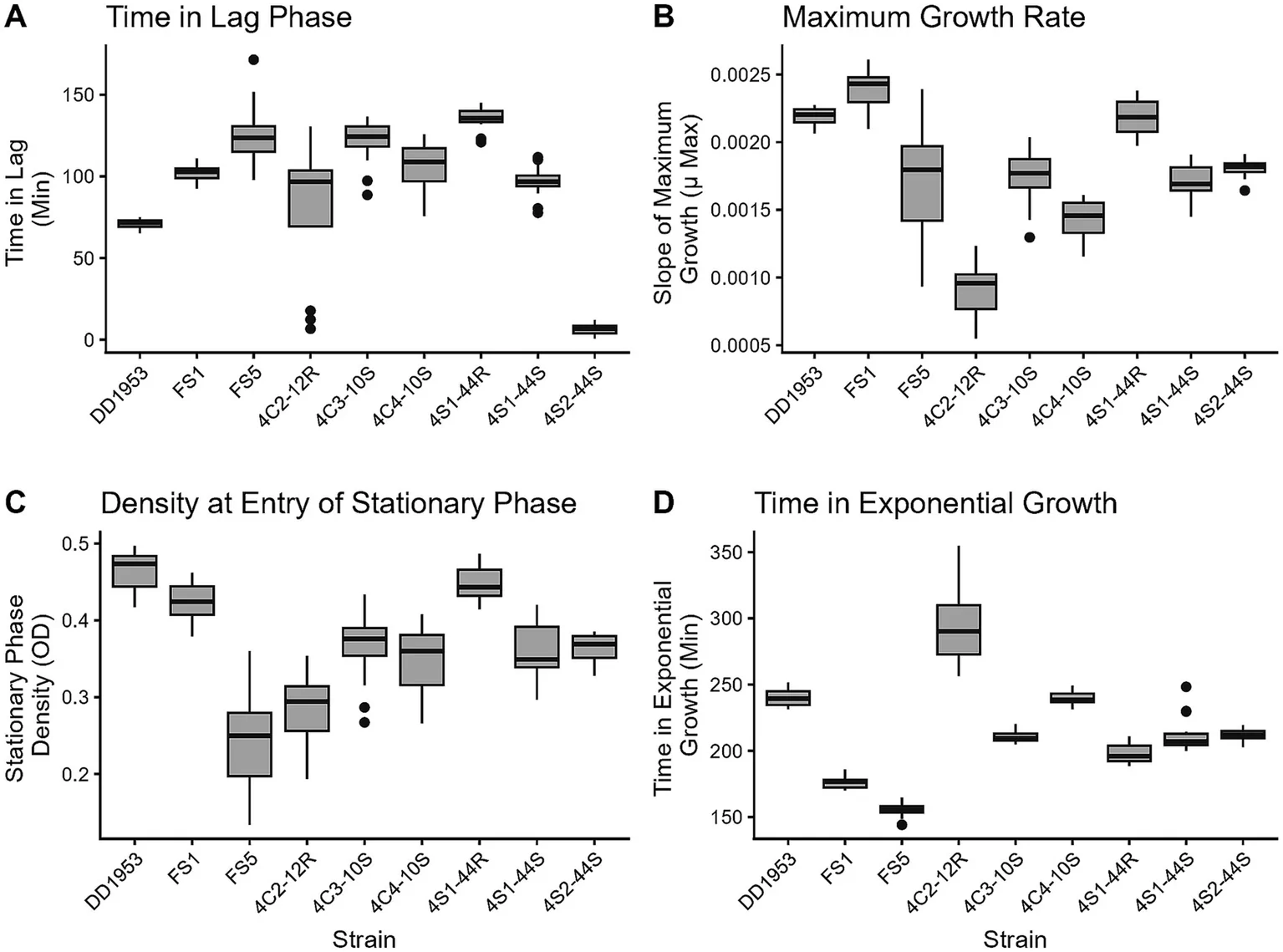
Boxplots of growth curve assay for ancestor and evolved strains. All strains were tracked in a 96-well plate assay for growth-related phenotypes. **(A)** Time spent in lag phase: no observable pattern for changes in time spent in lag phase was found for strains. **(B)** Maximum growth rate: counter to expectation, maximal growth rate did not decline for streptomycin-resistant strains FS1 or FS5 relative to DD1953 and then did not increase after evolution under serial passage conditions. In fact, *μ*max declined in flask-evolved strains relative to the antibiotic-resistant ancestors. **(C)** Population density at entry to stationary phase: the final density of experimental populations did not show a pattern after experimental evolution. **(D)** Time spent in exponential phase: the time spent in exponential growth significantly declined for streptomycin-resistant mutants FS1 and FS5 relative to the DD1953 ancestor. Following *in vitro* evolution, all evolved strains showed significant increases in the time spent in exponential growth, with values approaching those of DD1953. To our knowledge, selection for this phenotype has not been previously described relating to antibiotic resistance. Statistical comparisons: Pairwise *t*-tests with Bonferroni correction (Sokal and Rohlf, 1995).

We did, however, find strong support for directional selection on the amount of time spent in exponential growth across all evolved strains regardless of *in vitro* method, concentration of streptomycin, or type or number of mutations identified in evolved populations. The physiological & ecological reasons for extended time in exponential growth and how such a phenotype may increase rather than hinder fitness are well-known (Enjalbert et al., 2015; See Dean and Dykhuizen, 2026 for extended discussion). Changes in the time of exponential growth can be adaptive when mean growth rates are lower or when there are changes to the diauxic shift to a secondary energy source (for *E. coli* growing on glucose, this is typically acetate). Our results indicate that regardless of experimental condition, selection consistently acted upon the time in exponential growth. FS1 and FS5, the unevolved strains containing new mutations giving rise to streptomycin resistance, have a normal growth rate but a shortened time in exponential growth. In the strains evolved from FS1 and FS5, the time in exponential growth is similar to the original DD1953 strain, strongly suggesting that selection acted upon this phenotype and not protein synthesis rates as previously observed.

In order to better understand these results, a functional protein synthesis assay was conducted. Rather than indicating an increase in the speed and/or accuracy in producing functional proteins (particularly after compensatory mutations), the rate of synthesis of functional proteins for all evolved strains was *lower* than the streptomycin resistant ancestors in the presence of streptomycin (Figure 2). This indicates that the rate of protein synthesis, long thought to be the phenotype most affected by streptomycin resistance, may not tell the entire story of streptomycin-resistance adaptation in bacteria.

**Figure 2:**
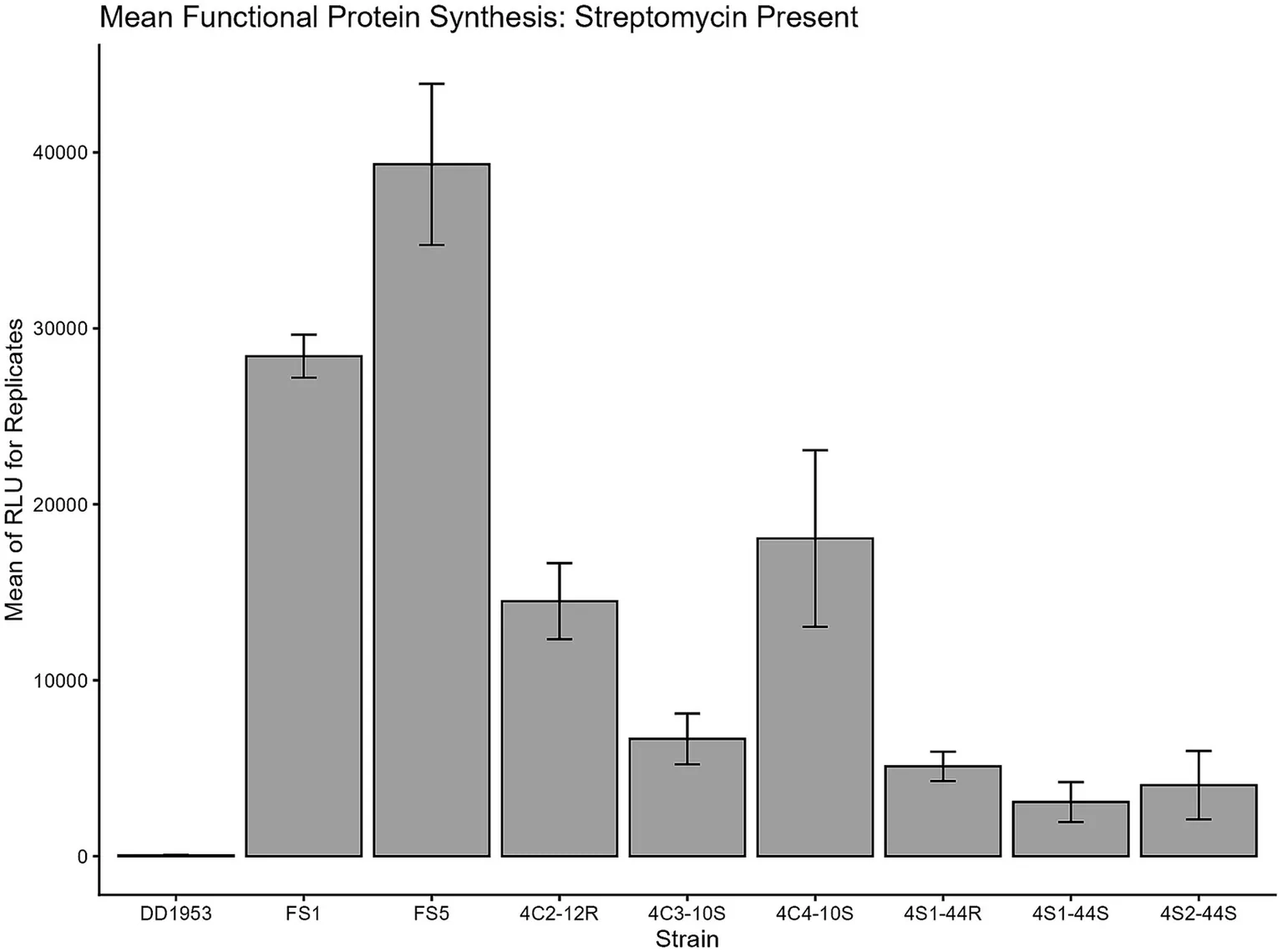
Mean functional protein synthesis with streptomycin present. A functional protein synthesis assay was developed that quantified the amount of functional firefly luciferase protein produced in one hour. Quantities taken using relative light units in a luminometer. Contrary to expectation, evolved strains had lower rates of function protein production than streptomycin resistant ancestors, FS1 or FS5. The evolved strain with the highest mean functional protein production (and largest confidence interval) is 4C4-10S, which evolved *without* streptomycin present in the chemostat. Error bars indicate 95% CI. See Supplementary material for assay conditions and protocol.

As a control, we also performed the protein synthesis assay under strep free conditions. Under these conditions, DD1953, the strep-sensitive ancestor, produced functional protein at a rate 2,372X higher than in the presence of streptomycin (Supplementary Figure S1). For evolved strains, the ratio of functional protein measured with and without streptomycin present was about 1. Two of three flask evolved strains had increases in functional protein synthesis when streptomycin was absent, but this is an environmental condition in which they did not evolve and the rate of protein synthesis was still approximately two orders of magnitude lower than in wild type. Additional data for the protein synthesis assays is in the Supplementary material.

## Discussion

The results presented here represent a mixture of the expected and unexpected and lead us to necessarily reassess our understanding of adaptation after antibiotic resistance.

### Experimental observations

We observed the fitness losses generally associated with antibiotic resistance and with resistance to streptomycin in particular (Björkman et al., 1999). Following resistance, we observe compensatory mutations in the *rpsD* and *rpsE* genes when post-resistance strains are evolved under serial transfer conditions. These compensatory mutations have been associated with increased ribosomal translation rates (Björkman et al., 1999). However, when evolved under chemostat conditions, we do not see these mutations but rather changes in the *galS* system, *regardless of the amount of streptomycin in the environment*. Similar changes in *galS* were observed in *E.coli* growing under glucose limited conditions in chemostats (Notley-McRobb and Ferenci, 1999), but in that case the strain of *E. coli* used, BW2952 (Genbank Accession CP001396), was not resistant to streptomycin. Given that both the serial transfer and chemostat experiments all used the same media and limiting glucose concentration, these mutations are most likely not related to carbon source as observed previously (Paulander et al., 2009), but rather to the specific environmental conditions differing between chemostats and flasks.

When we quantify the change in protein production across ribosomes, we find that compensatory *rpsD*/*rpsE* mutations lead to *lower* protein translation rates when streptomycin is present (Figure 2), but that protein synthesis is higher in the absence of streptomycin, even when the strain evolved only with antibiotic in the environment (Supplementary Figure S1). Such results are counter to the general understanding of compensatory evolution and lead us to conclude that compensatory evolution following antibiotic resistance is not universal.

### Evolutionary considerations and theoretical implications

All strains increased in relative fitness (Table 2), leading us to reasonably conclude that selection is at work. An investigation into phenotypic change in the higher fitness evolved strains indicates that the trait of interest is likely time in exponential growth (Figure 1). To our knowledge, neither ribosomal changes at *rpsD*/*rpsE* nor glucose uptake changes involving *galS* have been previously associated with changes to time in exponential growth.

This finding does not, however, explain our results in an evolutionary context nor does it explain why many antibiotic-resistant strains do not revert to sensitivity after the pressure for resistance is removed. In order to offer a hypothesis explaining our results within the framework of resistance/susceptibility, we believe it is necessary to consider both the evolvability (Wagner and Altenberg, 1996; Fragata et al., 2019) and the fitness landscapes (Payne and Wagner, 2019) of our strains *in the environments tested*. Here, we mean evolvability in the sense of Fragata et al. (2019), the ability of a population to produce variants that can be acted upon by (positive) selection. In chemostats, the genetic changes (*galS*) were predictable *a priori* based upon previous results (Notley-McRobb and Ferenci, 1999) and the experimental conditions, even if the phenotypic changes (time in exponential growth) were not. Genotypic changes did not depend on the antibiotic resistance state of the genetic background and reflected adaptation relating to the limiting nutrient (in this case, glucose but any limiting resource would likely have similar effect) (Notley-McRobb and Ferenci, 1999; Warsi and Dykhuizen, 2017).

In flasks, the genotypic changes observed were not accessible via natural selection prior to streptomycin resistance. These mutations were, however, open to selection *after* the switch to a streptomycin laden environment, suggesting that the antibiotic resistance mutation not only allowed for the strain to survive in the new environment, but it also increased the evolvability of the strain in this new, rugged fitness landscape by opening up adaptive paths that did not exist previously. In doing so, post-resistance adaptation potential (i.e., the number of possible adaptive paths accessible by selection) increased. This would potentially make the success of any counter-selection regime, such as those often relied upon in order to control the spread of resistance, highly improbable because the fitness landscape is different and adaptive movement on the *new* landscape does not necessarily map to equivalent movement on the previous landscape, even if it is reestablished.

The addition of antibiotic changes the environment substantially, which also changes the adaptive landscape from the perspective of the bacteria. Strains that were high fitness in the strep-free environment cannot survive after the addition of streptomycin. This may be most evident in a genotype to fitness mapping: the antibiotic sensitive ancestor, DD1953, was high fitness in the absence of streptomycin however, the DD1953 strain has an effective fitness of 0 once enough streptomycin is added to the environment. In such strep-laden environments, FS1 and FS5, which are lower fitness in the antibiotic free environment, instantly take on higher fitness. The environmental change may reveal hidden genetic variation (Szamecz et al., 2014), offering FS1 and FS5 access to higher fitness optima via selection. Our data suggest that such higher fitness peaks (Wright, 1956) would be related to the time spent in exponential growth phenotype. Importantly, our results indicate that *how* strains would access these peaks differ based upon their specific *in vitro* environments, with chemostats having an adaptive path similar to that available to strep-sensitive populations and batch cultures having an adaptive path contingent upon the strep-resistance mechanism, which was not available to strep-sensitive strains. In this way, phenotype and environment would conspire to increase evolvability in our streptomycin resistant strains in both expected and unexpected ways.

The increase in the number of possible adaptive paths for antibiotic resistant strains may provide a hypothesis to explain why selection against antibiotic resistance has proven so unpredictable. Populations at global optima, such as antibiotic sensitive strains, are likely to have inherent phenotypic compromises (Roff and Fairbairn, 2007). For a large population with sizeable genetic diversity, environmentally mediated release from a global optimum can be expected to allow for directional selection for any variety of previously inaccessible phenotypes. Such a shift and change in accessibility would reflect an increase in variability (Payne and Wagner, 2019). Antibiotic resistance represents a specialized case of just such an environmentally mediated release whereby the effect upon selection is particularly strong, with high fitness genotypes suddenly becoming lethal.

If evolvability does increase post-resistance, antibiotic rich environments may act as a mechanism that releases populations from the fitness peaks upon which they had been stranded and may provide additional paths with which they could then explore a newly formed fitness landscape. By changing allelic frequencies while also potentially simplifying rough adaptive landscapes, the environmental shift could increase variability and, therefore, evolvability.

Another way to potentially interpret our results is through the lens of Wright’s concept of a “shifting balance” (Wright, 1931, 1982). Shifting balance proposes that a species may cross valleys in a rugged fitness landscape through the combined effect of genetic drift and subsequent natural selection. Here, we posit individual populations crossing fitness valleys not by drift and selection, but by the restructuring of the landscape via an environmental shift (antibiotic) followed by specific selection for increased fitnesses based upon those new landscapes. Such selection for shifting peaks would explain the observed fitness increases in antibiotic resistant strains as well as the low possibility for counter-selection being a viable strategy for antibiotic sensitivity after the fact, at least in our condition of streptomycin resistance via a *de novo* single nucleotide mutation. Whether our experimental conditions prove to be specific or generalizable is not known at this time.

As early as 1975, H. W. Smith warned against the idea of combating established antibiotic resistance by expecting resistant strains to select for susceptibility simply by removing the antibiotic from the environment (Smith, 1975). After some tough lessons we have learned that reversing resistance will not be quite as simple as hoped (see Andersson and Hughes, 2010 for a review). More recently, however, new approaches have begun to show promise in reversing resistance, such as in some phage treatments for drug resistant bacterial populations (Chan et al., 2016). This study suggests that undoing what has been done may be even more complicated yet: switches in environments may increase the adaptive potential of resistant populations and allow for increases in fitness that cannot be predicted *a priori*.

## Data Availability

The original contributions presented in the study are publicly available. This data can be found here: NCBI GenBank, BioProject PRJNA1499252.
